# Amphibian Chytrid Fungus in Madagascar neither Shows Widespread Presence nor Signs of Certain Establishment

**DOI:** 10.1371/journal.pone.0139172

**Published:** 2015-10-14

**Authors:** Jonathan E. Kolby, Lee F. Skerratt

**Affiliations:** One Health Research Group, College of Public Health, Medical, and Veterinary Sciences, James Cook University, Townsville, Queensland, Australia; University of South Dakota, UNITED STATES

## Abstract

The global spread of amphibian chytrid fungus (*Batrachochytrium dendrobatidis*, *Bd*) is associated with amphibian mass mortality, population decline, and extinction. Over the past decade, concern has been expressed for the potential introduction of *Bd* to Madagascar, a global hotspot of amphibian biodiversity. Following years without detection, widespread *Bd* presence in Madagascar has now been reported (Bletz et al. 2015a), raising international conservation concern. Before reacting to this finding with a significant management response, the accuracy and context of the data warrant cautious review. Re-examination of a 10-year dataset together with results from more recent surveillance (Kolby et al. 2015) does not yet demonstrate widespread *Bd* presence. Detection of *Bd* at “positive” locations in Madagascar has been inconsistent for unknown reasons. Whether *Bd* is established in Madagascar (i.e. populations are self-sustaining) or instead requires continued introduction to persist also remains uncertain. The deployment of emergency conservation rescue initiatives is expected to target areas where the distribution of *Bd* and the risk of chytridiomycosis endangering amphibians is believed to overlap. Thus, erroneous description of *Bd* presence would misdirect limited conservation resources. Standardized surveillance and confirmatory surveys are now imperative to reliably characterize the distribution, potential spread, virulence and overall risk of *Bd* to amphibians in Madagascar.

## Introduction

Two recent papers provide conflicting results on whether the globally emerging amphibian chytrid fungus *Batrachochytrium dendrobatidis* (*Bd*) is now widespread and established (self-sustaining populations) in Madagascar [1,2]. Presence of *Bd* has been linked to a growing number of disease-driven amphibian mortality events and population declines since its discovery [3,4]. The global origin of *Bd*, timeline of first emergence, and reason for contemporary lethality are still unknown, although it has been present for thousands of years [5]. Despite its historic occupation in some areas and continued spread to others, certain regions appear to have remained free of *Bd* and disease-associated amphibian decline until only very recently, most notably Madagascar—a global hotspot of amphibian biodiversity.

A series of proactive *Bd* surveillance efforts performed prior to 2010 found the pathogen to be absent in Madagascar (0% estimated prevalence with high confidence) [6–8]. Assuming *Bd* absence based on these data and considering the threat of introduction, the Madagascar Chytrid Emergency Cell was established in 2010 along with the development of the National Monitoring Plan to administer biannual surveys for *Bd* presence, performed at eight nationally distributed locations to facilitate early detection and a rapid response [9]. Soon thereafter, a first call to arms was raised when amphibians collected from the wild in Madagascar and exported to the USA pet trade in Feb 2012 tested positive for *Bd*, suggesting presence and potentially recent introduction to Madagascar [10], as no other detections had previously been confirmed.

Nearly three years later, Bletz et al. [1] now report widespread *Bd* presence in wild amphibian communities in Madagascar. Surveillance efforts described by Bletz et al. [1] span nearly a decade (2005–2014), involved the sampling of 4,155 amphibians, and were performed at 52 survey sites across the country. *Bd* was detected at 10 sites, from five locations (*i*.*e*. multiple sites tested positive at some locations, *e*.*g*. two separate *Bd*-positive stream sites within a national park). Infection prevalence reported from these sites was generally low, but reached up to 100% on occasion. Accordingly, this announcement of seemingly sudden widespread *Bd* distribution together with unpredictable high prevalence of infection ignited considerable alarm among the conservation community, as it suggests that catastrophic disease-associated decline in Malagasy amphibian biodiversity is now immediately possible.

One month following the final *Bd*-positive survey described by Bletz et al. [1] from Jan 2014, a highly intensive national surveillance program did not detect *Bd* presence in samples collected at 47 survey sites distributed throughout the country [2]. This sampling effort included 508 amphibians swabbed for *Bd* and 68 *Bd* eDNA water filter samples processed to detect the pathogen in amphibian habitats. Locations surveyed by Kolby et al. [2] included 3/5 *Bd*-positive regions described by Bletz et al. [1]. Although Bletz et al. suggested *Bd* detection is greater in the cooler dry season, this pathogen has also been detected in Madagascar during the warmer wet season and our lack of detection at “positive” locations is curious, especially since our methods incorporated multiple techniques to increase sensitivity and power of detection. Thus, field survey results presented by Kolby et al. [2] strongly conflict with the assertion that *Bd* is widely distributed in Madagascar.

Before significant conservation management decisions are developed based on the distribution of *Bd* described by Bletz et al. [1], it is important to consider and resolve the disparity in results provided by Kolby et al. [2]. Three main plausible explanations spring to mind: 1. the studies varied significantly in diagnostic sensitivity and specificity, 2. the amphibian chytrid fungus detected by PCR by Bletz et al. [1] and Kolby et al. [2] behaves differently to the *Bd* detected elsewhere in the world, and 3. non-*Bd* material is reacting with *Bd* primers and falsely suggesting its presence in Madagascar. Bletz et al. [1] partially addressed the latter hypothesis by conducting a lineage-specific PCR that suggested some of the positive samples were molecularly similar to the global pandemic *Bd* lineage, but histological confirmation of infection with *Bd* in Malagasy amphibians still does not yet exist. In considering hypothesis 1, variation in accuracy, as a possible explanation for the discordance among survey results, amphibian sampling efforts performed from 2005–2014 demonstrated an overall lack of standardization in survey design, including: the selection of locations sampled, sample collection and storage methods, and laboratory diagnostic techniques. Accuracy of the qPCR test for *Bd* detection has been shown to vary according to both field sampling and laboratory diagnostic methods [11] and Bletz et al. [12] further demonstrate that employing different DNA extraction methods will affect the detection of *Bd* from skin swabs. Given the aforementioned variation in field protocols and laboratory techniques, each study has introduced additional unique variation in diagnostic sensitivity and specificity over the 10 years of surveys. Thus, some unknown cumulative degree of uncertainty is now embedded within the description of the current presence and distribution of *Bd* in Madagascar.

Two anomalies in the data specifically deserve attention: inconsistent detection at sites reported as *Bd*-positive and dramatic fluctuation in measured *Bd* prevalence. Detection of *Bd* at specific survey sites was infrequently followed by subsequent detection (Table 1). For example, *Bd* was last detected at Makay and Ankarafantsika 3–4 yrs ago, in 2011 and 2012, respectively, and recent confirmatory survey efforts at both sites produced only negative results. If these data reflect true *Bd* absence following earlier detections, then it is plausible that *Bd* failed to become established at these locations and develop self-sustaining populations. These observations now introduce uncertainty regarding the current presence of *Bd* at most sites in Madagascar reported as “*Bd*-positive”. This is because 8/10 of these sites were classified as “*Bd*-positive” based on single detection events (Table 2), where the assumption of *Bd* establishment may likewise be spurious. This sporadic pattern of *Bd* detection also raises question as to the manner in which *Bd* survey results are commonly communicated; do sites of *Bd* detection followed only by lack of detection warrant a "*Bd*-positive" label and permanent loss of freedom of disease status? The World Organisation for Animal Health (OIE) Aquatic Animal Health Code (Chapter 8.1: Infection with *Batrachochytrium dendrobatidis*) states that a country where *Bd* has been detected can be reclassified as “free from infection with *B*. *dendrobatidis*” if targeted surveillance has been in place for at least two consecutive years without detection [13]. If this approach was to be applied at the field site level within Madagascar, and elsewhere, it would substantially change the manner in which researchers populate *Bd* distribution maps—at present, even a single *Bd*-positive record is frequently used to symbolize *Bd* persistence at that location, with or without subsequent surveillance.

**Table 1:** Timeline of *Batrachochytrium dendrobatidis* (*Bd*) records of detection at affected sites in Madagascar from 2005–2014 as reported by Bletz et al. (2015a) and Kolby et al. (2015).

Location	Site	Total sample	2005–2009	2010	2011	2012	2013	2014A	2014B
Ankaratra	Tavolotara	167 (18)	**–**	–		X (unk)	X (18)		–
Ankaratra	Ambatolampy	64 (7)	–				X (7)		–
Ankaratra	Ambohimirandrana	42 (8)		–			X (8)		–
Ankarafantsika	Andranofasika	150 (unk)			–	X (unk)	–		–
Makay	Andranovinily	164 (3)		X (3)	–		–		
Makay	Beroroha	209 (1)			X (1)		–		
Ranomafana	Vatoharanana	117 (15)					X (15)	–	–
Ranomafana	Valohoaka	43 (1)						X (1)	–
Antoetra	Fohisokina	150 (unk)			–	X (unk)			
Antoetra	Soamazaka	9 (1)						X (1)	
Total		1115 (54)	0	3	1	unk	48	2	0

Surveys with *Bd* detection marked by "X" and those with only negative results marked by "–". The number of *Bd*-positive amphibians detected in each survey is presented in parenthesis, except where samples were pooled for analysis and the number of *Bd*-positive animals remains unknown (unk). Where multiple surveys were performed at a site within the same year, data were combined. The column "2014A" represents surveys reported by Bletz et al. (2015a) and "2014B" represents surveys reported by Kolby et al. (2015)

**Table 2:** Number of surveys and detections for all sites in Madagascar reported as positive for the presence of *Batrachochytrium dendrobatidis* (*Bd*) by Bletz et al. (2015a) and the most recent survey results reported by Kolby et al. (2015).

Location	Site	sample size (*Bd*+)	1 survey 1 detection	2 surveys 1 detection	≥ 3 surveys 1 detection	2 surveys 2 detections	≥ 3 surveys 2 detections	≥ 3 surveys 3 detections	Feb-March 2014
Ankaratra	Tavolotara	167 (18)					**X**		–
Ankaratra	Ambatolampy	64 (7)		**X**					–
Ankaratra	Ambohimirandrana	42 (8)					**X**		–
Ankarafantsika	Andranofasika	150 (unk)			**X**				–
Makay	Andranovinily	164 (3)			**X**				**N/A**
Makay	Beroroha	209 (1)		**X**					**N/A**
Ranomafana	Vatoharanana	117 (15)		**X**					–
Ranomafana	Valohoaka	43 (1)	**X**						–
Antoetra	Fohisokina	150 (unk)			**X**				**N/A**
Antoetra	Soamazaka	9 (1)	**X**						**N/A**
Total		1115 (54)	2	3	3	0	2	0	0

The number of surveys performed at each location is expressed together with the number of those events that resulted in *Bd* detection, as reported by Bletz et al. (2015a). Cumulative number of amphibians sampled at each site by Bletz et al. (2015a) and number of *Bd*-positive animals detected (*Bd*+) is reported, except where samples were pooled for analysis and the number of *Bd*-positive animals is unknown (unk). The final column ("Feb-March 2014") represents the most recent survey results as reported by Kolby et al. (2015), from sampling at or near sites of previous *Bd* detection. Areas not surveyed by Kolby et al. (2015) are marked with "N/A"

Bletz et al. [1] mention that their long-term methodological inconsistencies place a limitation on data interpretation and,"… may confound time and season with detection method and could in part compromise the conclusions of the recent detection of *Bd* and seasonality trends." The exceptionally high *Bd* prevalence of 100% inferred from material collected at Ankaratra (site: Tavolotara) in Aug 2013, was both preceded and followed by complete lack of detections in Feb 2013, Dec 2013, and March 2014. Environmental conditions measured during the most recent survey at Ankaratra in March 2014 were favorable for *Bd* to thrive, with daytime air and water temperatures ranging from 12.4–15.9 C and 13.4–15.2 C, respectively. *Bd* mortality (i.e. death of the pathogen) does not normally occur until temperatures rise to 28 C and above [14], and even following death for any reason, its molecular presence will persist and extend detectability. In an aquatic habitat, *Bd* is likely to remain detectable year-round despite sometimes variable abundance, as has been observed in long-term water filtration surveys at affected wetlands [15]. Although the *Bd* in Madagascar might behave dramatically unlike that studied elsewhere, no evidence yet supports this hypothesis apart from variation in virulence observed among lineages [16]. Bletz et al. [1] hypothesize that this phenomenon at Ankaratra may be explained by a seasonal variation in *Bd* abundance caused by some unidentified factor and/or there was a changeover in the strain of *Bd* present (similar to hypothesis 2, above). Considering all available information, the law of parsimony instead suggests that samples from Aug 2013 were more likely compromised and involved some form of field or laboratory error (hypothesis 1).

In order to reduce aforementioned uncertainties, current and future *Bd* surveillance methods must be standardized to prevent continued challenges in field data interpretation and potential loss of conservation value produced by these efforts. Moving forward, variation in detection that may confound observed patterns of *Bd* presence can be minimized by establishing longer-term surveys at suspected *Bd*-positive sites and ensuring that samples are collected, stored, and analyzed following standardized established methods for *Bd*. Multiple complementary *Bd* sample collection and diagnostic techniques can also be incorporated into future surveys to increase the confidence of results, *e*.*g*. collection of water samples for eDNA analysis at aquatic habitats where amphibian are simultaneously swabbed for Bd [2], and histological confirmation of *Bd* presence in an amphibian that tests positive by skin swab [17]. Water filtration can capture *Bd* particles shed from a host [15,18,19] and if detected, corroborate swab-based "*Bd*- positive" site characterizations in a highly cost-efficient manner. At the time of writing (August 2015), histological evidence of *Bd* infection in wild Malagasy amphibians still does not exist; such data would help prove that *Bd* presence is certainly causing the PCR-based detections and help rule out hypotheses 1 and 3, above. Further, fungal isolation should be performed and results verified by an OIE reference laboratory [17].

In conclusion, the uncertain distribution and potential impact of *Bd* presence in Madagascar requires additional investigation before accurate evaluations can be made. Standardized field surveillance methods and laboratory diagnostic techniques are needed to more carefully investigate the presence of *Bd* both at sites where it has and has not yet been detected. The former is necessary to build longer-term surveys that can assess whether or not Bd is established in Madagascar and the latter to monitor future *Bd* spread into potentially *Bd*-negative locations. These data can then be combined with those provided by Bletz et al. [1] and Kolby et al. [2] to more confidently assert whether *Bd* is truly present and widespread, especially in the absence of any obvious sign of disease. Still, many amphibian species in Madagascar have not been monitored in a long-term standardized fashion, and thus any *Bd*-associated decline may be occurring unnoticed. Therefore, population monitoring of key species likely to be affected warrants urgent establishment. The application of skeletochronology [20] and/or intensive mark-recapture surveys may shed light on survival patterns in amphibian communities where *Bd* has been identified or is suspected. The presence, distribution, virulence to native species and clade membership of *Bd* in Madagascar must be verified before its potential impact on Malagasy amphibians can be accurately predicted.
